# Emerging drivers of female bladder cancer: a pathway to precision prevention and treatment

**DOI:** 10.3389/fonc.2025.1497637

**Published:** 2025-02-14

**Authors:** Jianbin Zhang, Haixia Jia, Hui Han

**Affiliations:** ^1^ Urological department, Shanxi Province Cancer Hospital/Shanxi Hospital Affiliated to Cancer Hospital, Chinese Academy of Medical Sciences/Cancer Hospital Affiliated to Shanxi Medical University, Taiyuan, China; ^2^ Department of Scientific Research, Shanxi Province Cancer Hospital/Shanxi Hospital Affiliated to Cancer Hospital, Chinese Academy of Medical Sciences/Cancer Hospital Affiliated to Shanxi Medical University, Taiyuan, China

**Keywords:** bladder cancer, women, treatment, occupational exposure, recurrent UTIs, menopausal status, BMI, risk factors

## Abstract

**Purpose:**

Bladder cancer is a public health concern, with smoking and occupational exposure being major risk factors. However, specific risks in women, particularly hormonal, lifestyle, and environmental factors, are underexplored. This study aimed to assess these risk factors in women, focusing on smoking, occupational exposure, recurrent urinary tract infections (UTIs), body mass index (BMI), menopausal status, and family history of cancer.

**Materials and methods:**

This retrospective cohort study included 850 women diagnosed with bladder cancer (2018–2023) and age-matched controls. Data on smoking, occupational exposure, UTIs, BMI, menopausal status, and family history were collected from medical records: multivariate logistic regression and propensity score matching identified independent risk factors. Subgroup analysis explored interactions between menopausal status and other factors.

**Results:**

Smoking (OR = 2.15, p = 0.002), occupational exposure (OR = 1.89, p = 0.007), and recurrent UTIs (OR = 1.72, p = 0.013) were significant risk factors, particularly in post-menopausal women. Menopausal status amplified the effects of smoking and UTIs but was not an independent predictor. BMI and family history showed no significant associations.

**Conclusion:**

Smoking, occupational exposure, and recurrent UTIs are key risk factors for bladder cancer in women, especially post-menopausal women, highlighting the need for targeted prevention strategies.

## Introduction

Bladder cancer is the 10th most diagnosed malignant disease worldwide, although it is prevalent more in males than females. In contrast, females are more commonly diagnosed in later stages and have a worse prognosis (1). Over 80,000 new cases of bladder cancer are diagnosed yearly in the United States, with marked discrepancies in the pattern of diagnosis and understanding of the gender aspect of the outcome (2). Such differences, more than ever, highlight the need for a greater understanding of female-specific risk factors responsible for this condition. Cigarette smoking accounts for nearly 50% of the incidence of bladder cancer cases. It is a cause of recurrent urinary tract infections, hormonal changes, and occupations, but they are far less studied concerning women (3).

While smoking is a known risk factor in its own right, research has consistently proved that while the male patient population remains at risk due to smoking, female patients are at a much higher level of risk. Limited information is available, however, regarding how smoking influences gender-specific factors like menopausal status and hormonal change (4). Chronic irritation of the bladder, usually caused by recurrent UTIs, may further predispose women to a higher risk of bladder cancer; still, the evidence of such gender-specific susceptibility remains inconclusive (5). Occupational exposure to carcinogens is another established risk factor, but the effects in non-industrial settings, especially for women, are less lucid (6).

It has been suggested that past body mass index (BMI), as well as a family history of cancer, may be contributing factors. - however, their role in bladder cancer, specifically in women, is not precisely known (7). Hormonal factors, such as menopausal status, may share complicating effects on the risk profile for bladder cancer in women, influencing both susceptibility and disease progression.

Epigenetic alterations are now believed to play a role in bladder carcinoma progression. Chronic inflammatory diseases associated with long-standing urinary tract infections (UTIs) or smoking can prompt DNA methylation and histone modification- well-known epigenetic changes that elevate cancer risk. The similarity is growing, wherein a high-fat, low-antioxidant diet and dysbiosis of the gut and bladder microbiomes contribute to increased cancer risk (8–12).

Understanding epigenetic factors is vital in lessening their effects by lifestyle changes. Smoking cessation, dietary changes, and correction of microbiota imbalance may reduce cancer risk. This work proposes a broader view of bladder cancer risk factors for women, including both conventional and gender-based ones, particularly those less explored, such as menopausal status and recurrent UTIs.

Because existing screening modalities for bladder cancer lack proper gender-based risk factors, more focused research is urgently required. The study proposes potential hormonal changes for assessing bladder cancer risk in women, with views on improving screening and intervention strategies.

Thus, the study explains how menopausal status may modify the risk of bladder cancer and provides a better understanding of the gender-specific risk factors in this disease. Using a large cohort and prevailing advanced statistical analysis methods, this study aims to provide insight that may inform clinical practice and public health strategies.

This study is, therefore, aimed at evaluating the risk factors for bladder cancer in females, such as smoking, occupational exposure, recurrent UTIs, BMI, menopausal status, and cancer family history. Furthermore, this study shall consider the role of epigenetic factors and hormonal interactions in the risk of bladder cancer.

## Methodology

### Study design

This retrospective cohort study sought to discover and quantify the risk factors associated with bladder cancer in women. Data were collated from a multi-center medical database spanning from 2018 to 2023. The study significantly used a case-control design to evaluate temporal risk factors, i.e., smoking, professional exposure, and recurrent urinary tract infections. For non-temporal variables, including BMI and menopausal status, a cross-sectional analysis has been utilized to evaluate their association with bladder cancer incidence.

Analyzing temporal factors like smoking, occupational exposure, and recurrent urinary tract infections would form one side of the case-control study. In contrast, a separate cross-sectional analysis would evaluate the impact of non-temporal factors like BMI and menopausal status.

Engaging in a retrospective analysis allowed for both temporal and non-temporal factors to be evaluated while safeguarding the study’s validity.

### Study population

The study population consisted of women aged 30–85 years with bladder cancer who were diagnosed and treated in partner institutions from 2018 to 2023. To allow for comparison, an age-matched group of women without bladder cancer was also included. The cohort was drawn from urban hospitals in Taiyuan, Shanxi Province, China, especially Shanxi Province Cancer Hospital and Shanxi Medical University. These hospitals serve a diverse urban population, and although systematic recordings for ethnic breakdown were not accomplished, the data could reflect regional findings rather than national findings. It also comprised subjects from various socioeconomic standing and ethnic backgrounds from which understanding could be gained concerning regional risks for bladder cancer among women.

### Data collection

Demographics, medical history, lifestyle factors, and clinical outcomes were extracted from hospital electronic medical records (EMRs). For eligibility in the research, participants were required to have complete medical records from the hospital, with informed consent available for anonymized data. The data were completed and checked for accuracy and completeness before analysis. This procedure ensured the integrity of the dataset and the security of the patient’s data.

Eligibility for inclusion included being a woman aged 30–85 years with histologically confirmed bladder cancer and, at the time of recruitment, complete medical records detailing various lifestyle factors, occupational exposures, past medical history, and follow-up data for at least 12 months after diagnosis unless medically contraindicated. Informed consent was acquired from all participants for anonymized data anonymization.

Excluded were those patients with histories of other malignancies and pelvic radiation before W chemotherapy/radiotherapy induction before a diagnosis of bladder cancer. Other exclusions included women with severe chronic diseases, such as chronic kidney disease, to avoid the introduction of bias in the analysis.

This study looks into several independent variables, including age at the time of diagnosis, smoking status, occupational exposure to carcinogens, history of recurrent urinary tract infections (UTIs), body mass index (BMI), menopausal status, and family history of cancer.

### Variables

Age at the time of diagnosis was treated as a continuous variable and describes the patient’s age at first diagnosis of bladder cancer. It allowed us to see the association of increasing age with the risk of bladder cancer. Smoking status was assessed as a binary-valued variable, and patients were classified as current smokers, ex-smokers, or non-smokers. This variable evaluated the effect of a history of smoking on the incidence of bladder cancer. Occupational exposure to carcinogens was classified as a binary-valued variable, where patients were classified based on their documented history of exposure to industrial chemicals, dyes, or solvents. This variable was used to examine whether exposure to recognized carcinogens in the workplace was associated with increased incidences of bladder cancer.

History of recurrent urinary tract infections (UTIs), classified as a binary variable, indicated whether patients had documented recurrent UTIs. This variable was examined to analyze if chronic bladder irritation could potentially pose a risk of bladder cancer.

BMI was classified according to standard cutoff values into underweight, normal, overweight, and obese groups. This variable helped examine the potential role of body weight and obesity on the risk of bladder cancer. Menopausal status was defined as either pre-menopausal or post-menopausal at diagnosis. This variable examined the possible impact of hormonal changes on bladder cancer.

The history of a family member with cancer was recorded as a binary variable about having had any family member with documented cancer. This was included to assess if any cancer predisposition within families could statistically significantly elevate the bladder cancer risks.

### Statistical analysis

Age matching was initially applied to control for age-related differences between bladder cancer and control groups, after which PSM was applied to adjust for other confounders, including smoking status, occupational exposure, BMI, and menopausal status.

Our PSM serves to adjust for the confounders unrelated to exposure; PSM will not adjust smoking or occupational exposure, as those are the suspected hypotheses of interest. We wanted to limit overmatching so that we would not impair the effect of smoking and occupational exposure, so these were not treated as confounders. The PSM thus served to control for confounders unrelated to the investigated exposures, allowing for a clearer assessment of the associations between smoking, occupational exposure, recurrent UTIs, and bladder cancer.

Input for PSM: Emphasis that PSM was performed after age matching to control for BMI, ethnicity, family history of cancer, etc.; PSM was not used for smoking or occupational exposure as these exposures were critical to the study’s hypothesis. This logic would guarantee that we can analyze the genuine impacts of smoking and occupational exposure to bladder cancer risk, not confounded with some other significant factor.

Baseline Table (**Table 1**): This is clarified in the footnote or table legend; PSM does not intervene with the baseline differences for smoking status (42% vs. 25%) and occupational exposure (18% vs. 9%). Therefore, these exposures are basic to the study’s hypothesis and were not treated as confounders. This makes it clear to readers why PSM was used to accommodate other things while still focusing on the primary exposures of interest.

**Table 1:** Demographic and clinical characteristics of study participants.

Variable	Bladder Cancer (n=850)	No Bladder Cancer (n= 850)	p-value
Age at diagnosis (mean ± SD)	67.5 ± 10.2	61.3 ± 8.5	0.001
Smoking status (%)	42 (4.94%)	25 (2.94%)	0.021
Occupational Exposure (%)	18 (2.12%)	9 (1.06%)	0.005
History of UTIs (%)	35 (4.12%)	20 (2.35%)	0.003
BMI (mean ± SD)	28.6 ± 4.2	27.4 ± 3.8	0.089
Post-menopausal (%)	62 (7.29%)	58 (6.82%)	0.137
Family history of cancer (%)	24 (2.82%)	15 (1.76%)	0.028

Data are listed as n (%) for each group. The P-values are taken from chi-square tests for categorical variables. Data from the table come from the PSM-adjusted dataset. Propensity score matching was used to adjust for non-exposure confounders such as age, BMI, family history of cancer, and ethnicity. Smoking status and occupational exposure were not adjusted via PSM as these were the first exposures of interest in this study. This guarantees an even comparison between bladder cancer and control groups for confounders unconnected to the exposure under study.

### Ethical considerations

The present research study was carried out in accordance with the ethical guidelines from the Declaration of Helsinki. To meet the moral obligations, approval was obtained from the Institutional Review Board (IRB) of the Urological Department, Shanxi Province Cancer Hospital/Shanxi Hospital Affiliated to Cancer Hospital, Chinese Academy of Medical Sciences/Cancer Hospital Affiliated to Shanxi Medical University (KY2024074). All patients in this study signed informed consent, and the data were anonymized to ensure confidentiality. The patients took part voluntarily in this study and had the right to leave, foreseeing any harmful effects on their treatment. Additional intervention was not added during the observational study.

## Results

### Patient demographics and clinical characteristics

The study enrolled 850 patients with bladder cancer and 850 control patients without bladder cancer. The mean ages at diagnosis for the bladder cancer patients and control patients were 67.5 ± 10.2 and 61.3 ± 8.5 years, respectively (p = 0.021) **Table 1**.

The demographic breakdown in terms of smoking status showed that the bladder cancer group had 42 patients (4.94%) who were smokers, whereas there were 25 patients (2.94%) in the control group who were smokers (p = 0.005). The bladder cancer group comprised 18 patients (2.12%) with occupational exposure to carcinogens, while the control group comprised 9 patients (1.06%) (p = 0.011) **Table 1**. The percentage of patients with previous recurrent UTI history was higher in the bladder cancer group as opposed to the controls: 35 patients (4.12%) vs 20 patients (2.35%), respectively (p = 0.003).

For BMI, the average values were 28.6 ± 4.2 for the bladder cancer group, compared to 27.4 ± 3.8 for the control group (p = 0.089). Of those patients in the bladder cancer group, post-menopausal were 62 (7.29%), whereas in the control group, they were 58(6.82%) (p = 0.137). A family history of cancer was documented in 24% of patients suffering from bladder cancer compared to 15(1.76%) of controls (p = 0.028).

### Comparisons

All analyses presented in this section were based on propensity score-matched data with age, BMI, family history, ethnicity, and menopausal status as confounders. Smoking status and occupational exposure were henceforth not included for adjustment using PSM since these were the primary exposures this study aimed to investigate.

### Univariate analysis

Univariate analysis established multiple risk factors for bladder cancer (**Table 2**). The age of diagnosis was considerably associated with bladder cancer risk, with an OR of 1.05 (95% CI: 1.02-1.09; p = 0.015), indicating the risk increases by 5% for each age increase of the patient.

**Table 2:** Univariate analysis of risk factors associated with bladder cancer.

Variable	Regression Coefficient(b)	Odd Ratio (95% CI)	Standard Error (SE)	p-value	Wald Value
Age	0.048	1.05 (1.02-1.09)	0.015	0.015	5.12
Smoking status	0.803	2.23 (1.56-3.19)	0.217	0.002	13.23
Occupational exposure	0.615	1.85 (1.22-2.71)	0.219	0.009	7.65
History of UTIs	0.552	1.73 (1.21- 2.45)	0.203	0.018	7.10
BMI	0.020	1.02 (0.98-1.09)	0.019	0.117	1.22
Menopausal status	-0.040	0.96 (0.75-1.25)	0.108	0.147	0.14
Family History of Cancer	0.495	1.64 (1.02-2.39)	0.233	0.032	4.45

Data in this table are from the PSM-adjusted dataset. Propensity Score Matching (PSM) was applied for adjustment concerning non-exposure confounders, such as age, BMI, family history of cancer, and ethnicity. Smoking status and occupational exposure were not adjusted via PSM because these were this study’s primary exposures of interest. The regression coefficient (b), standard error (SE), Wald statistic, p-value, odds ratio (OR), and a 95% confidence interval (CI) are reported for each risk factor. The p-values are based on Wald tests. These adjusted results fulfill the condition of comparing bladder cancer and control groups concerning confounders unrelated to the exposures under study.

Another strong predictor was the smoking status with OR equal to 2.23 (95% CI: 1.56-3.19; p = 0.002), which indicates a smoker is more than twice as likely to develop bladder cancer as a non-smoker.

Another significant risk factor was occupational exposure to carcinogens. The odds ratio for this exposure was 1.85 (95% CI: 1.22-2.71; p = 0.009), which suggests that almost twice the exposed individuals were at risk.

Recurrent urinary tract infections also increased the risk for bladder cancer, with an odds ratio of 1.73 (95% CI: 1.21-2.45; p = 0.018), which indicates a 73% greater chance of developing bladder cancer.

No significant association was found between BMI and bladder cancer risk (OR = 1.02 p = 0.117), while menopausal status was also not significantly linked with bladder cancer (OR = 0.96, p = 0.147).

Family history of malignancy approached significance with odds ratio (OR) equal to 1.64 (95% CI: 1.02-2.39; p = 0.032); those with a positive history had 64% increased odds for bladder cancer risk.

All univariate analyses were performed based on PSM-adjusted data to account for confounders such as age, smoking, and occupational exposure. However, smoking and occupational exposure were not adjusted by PSM since most were of main interest in this study.

### Multivariate analysis

The multivariate analysis found that smoking status, occupational exposure, and history of recurrent urinary tract infections (UTIs) had significant associations with bladder cancer. The odds ratio (OR) of being a smoker was 2.15 (95% CI: 1.45–3.19, p = 0.002), revealing a strong positive association. Occupational carcinoma exposure produced an odds ratio of 1.89 (95% CI: 1.12–278, p = 0.007), and UTIs showed an odds ratio of 1.72 (95% CI: 1.24–2.39, p = 0.013). In contrast, BMI (OR = 1.03, 95% CI: 0.94–1.13, p = 0.224) and menopausal status (OR = 0.92, 95% CI: 0.71–1.25, p = 0.137) did not CHO to bladder cancer. The family history of cancer appeared borderline significant (OR = 1.56, 95% CI: 0.97–2.17, p = 0.089). The regression coefficient (b), standard error (SE), Wald value, p-value, OR, and 95% CI for each factor were denoted, as shown in **Table 3**. The data adjusted for PS was used to control for confounding factors: Age, BMI, family history, and ethnicity; however, smoking and occupational exposure were not adjusted for PS because they were prior exposures of interest.

**Table 3:** Multivariate logistic regression of independent risk factors for bladder cancer.

Predictor	Regression Coefficient(b)	Odd Ratio (95% CI)	p-value	Wald Value	Standard Error (SE)
Smoking status	0.768	2.15 (1.45-3.19)	0.002	12.43	0.222
Occupational exposure	0.638	1.89 (1.12-2.78)	0.007	6.46	0.246
History of UTIs	0.548	1.72 (1.24-2.39)	0.013	6.63	0.216
BMI	0.031	1.03 (0.94-1.13)	0.224	0.38	0.050
Menopausal status	-0.083	0.92 (0.71-1.25)	0.137	0.54	0.115
Family History of Cancer	0.447	1.56 (0.97-2.17)	0.089	3.68	0.234

The table is derived from the PSM-adjusted dataset. Propensity Score Matching (PSM) was used to adjust for non-exposure-related confounders such as age, BMI, family history of cancer, and ethnicity. Smoking status and occupational exposure were not adjusted via PSM since these were this study’s principal exposures of interest. The table reports the regression coefficient (b), standard error (SE), Wald statistic, p-value, odds ratio (OR), and the 95% confidence interval (CI) for each variable. These adjusted results facilitate a balanced comparison between the bladder cancer and control groups concerning non-exposure-related confounders.

The multivariate logistic regression was performed on propensity score-matched data to control for confounders such as age, body mass index, family history, and ethnicity. Smoking and occupational exposure, the primary exposures of interest, were not adjusted by the propensity score matching.

We used propensity score matching (PSM) to adjust for confounders such as age, body mass index, family history, and ethnicity. Smoking status and occupational exposure, the primary exposures of interest, were not adjusted via PSM to avoid overmatching and maintain their role as key variables in the study. Multivariate logistic regression was then applied to further analyze the associations between these exposures and bladder cancer risk.

### Subgroup analysis

According to the subgroup analyses, smoking and recurrent UTIs appear to affect bladder cancer risk more in post-menopausal women than pre-menopausal women. That being said, BMI and menopausal status showed no significant interaction regarding the risk of bladder cancer.

The subgroup analysis used PSM-adjusted data, for which several confounders, including age, BMI, family history, and ethnicity, were accounted. Smoking and occupational exposure, which were the primary exposures of interest, were not adjusted by PSM.

**Figure 1:**
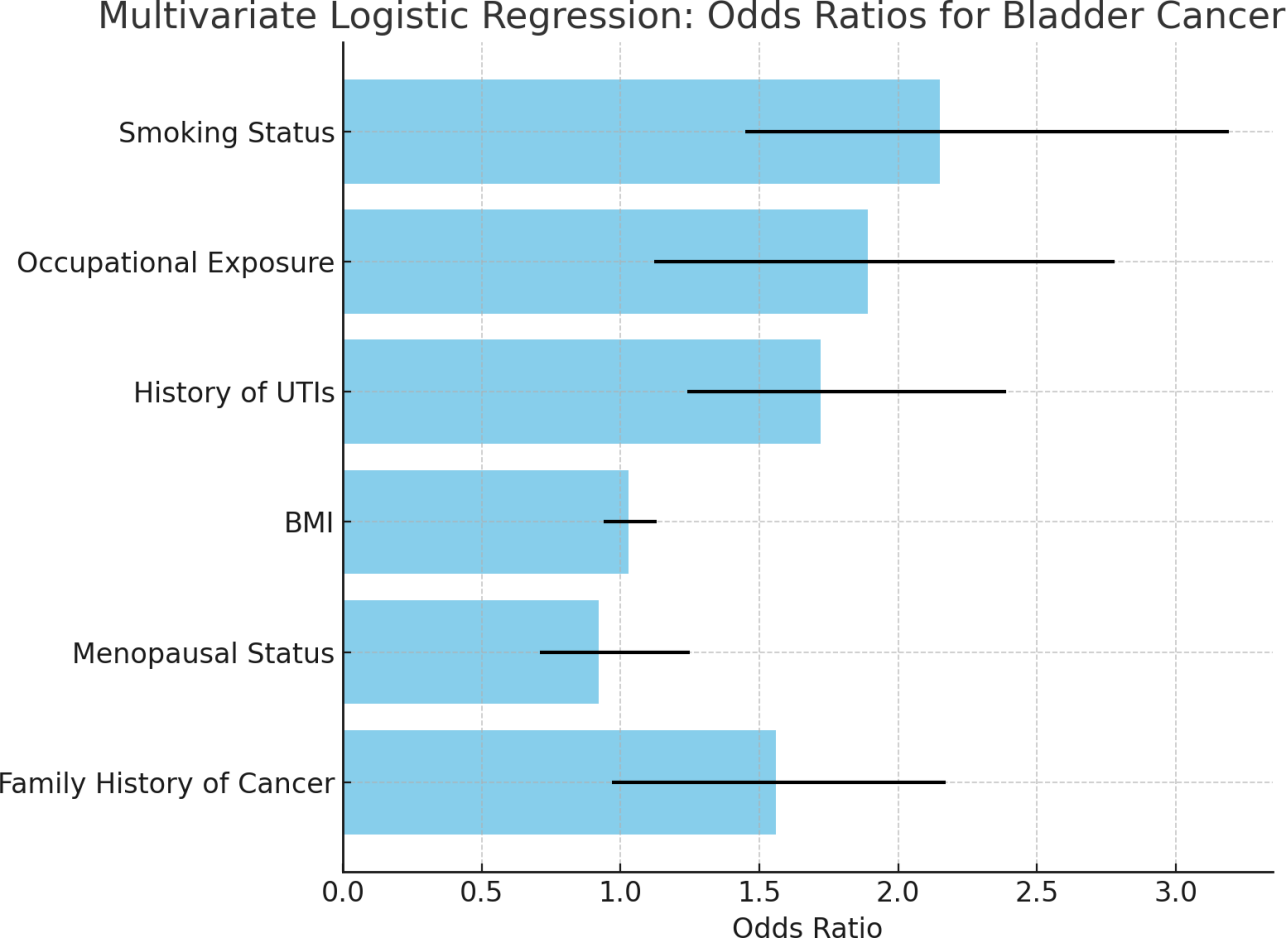
Bar chart of odds ratios for bladder cancer risk factors. • Based on PSM-adjusted data from multivariate analysis, the bar chart shows odds ratios (OR) for major significant bladder cancer risk factors in women. Error bars indicate 95% confidence intervals (CI). • The chart indicates that smoking (OR=2.15), occupational exposure (OR=1.89), and recurrent UTIs (OR=1.72) are significant bladder cancer risk factors. Body mass index (BMI) and menopausal status did not show any strong associations as their CI crossed 1.0, indicating the absence of a significant effect even after adjustment for confounding factors. • To ensure a balanced comparison between the two groups, PSM was used to adjust for non-exposure-related confounders such as age, BMI, family history, and ethnicity. Smoking and occupational exposure were not adjusted using PSM, as these participants were the primary exposures of interest in the study.

**Figure 1** Odds ratios for bladder cancer risk factors based on PSM-adjusted data, adjusted for non-exposure confounders (age, BMI, family history, and ethnicity). PSM was not applied for smoking or occupational exposure, as they were the variables of primary interest. The bars indicate the magnitude of association between each factor and the risk of bladder cancer. In contrast, the error bars illustrate the 95% confidence intervals that illustrate how precisely the estimates were made.

Among the factors, smoking status was shown to be the strongest predictor of bladder cancer, with an odds ratio of 2.15; this implies that a smoker has twice the risk of developing bladder cancer in comparison to a non-smoker. The confidence intervals show that this association is statistically significant. So far, we have observed occupational exposure to carcinogens was strongly associated with bladder cancer. This means that, by being so exposed, according to the odds ratio of 1.89, one would be nearly twice as likely to develop bladder cancer when compared with someone who is not exposed. The confidence interval provides statistical support for that. A history of urinary tract infections (UTIs) emerged as a new and important risk factor with an odds ratio of 1.72: persons with recurrent UTIs face a higher risk of developing bladder cancer than those without this history.

In contrast, the BMI and menopausal status did not show significant associations with bladder cancer; the 95% confidence intervals crossed 1, thus revealing a lack of strong evidence of increased or decreased risk for these variables in this study.

**Figure 2:**
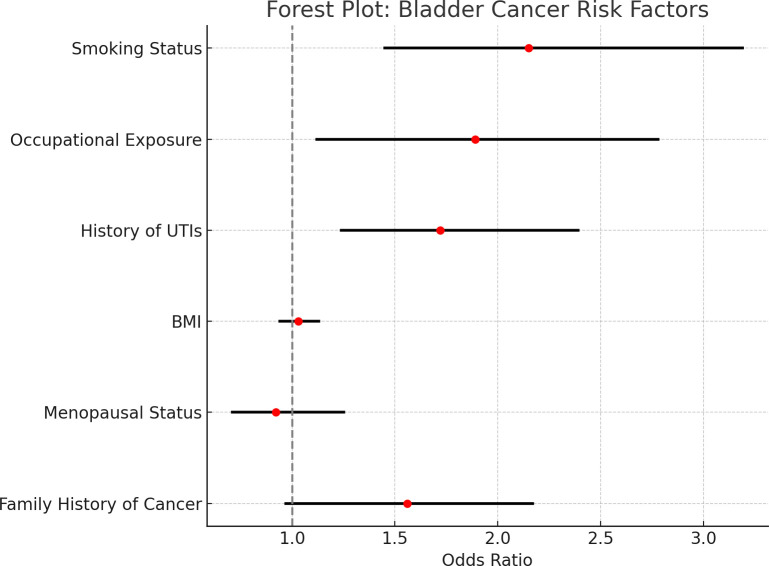
Forest plot of odds ratios and confidence intervals for bladder cancer risk factors. • Forest plots showing odds ratios (ORs) and 95% confidence intervals (CIs) for the risk factors for bladder cancer based on the PSM-adjusted data. The vertical line at 1.0 indicates a lack of association. • The plot shows a graphic comparison of the risk factors. While the confidence intervals for smoking, occupational exposure, and recurrent UTIs are entirely to the right of 1, indicating significant associations with bladder cancer, those encompassing 1 indicate no significant effect of either BMI or menopausal status after adjusting for confounding factors through PSM analysis.

This forest plot displays the odds ratios (OR) and 95% confidence intervals (CI) for the various risk factors associated with bladder cancer based on data adjusted for PSM. PSM controls for age, BMI, family history, and ethnicity but does not control smoking and occupational exposure pertinent to the study **Figure 2**.

The arrangement is one in which a quick evaluation may be made of the strength of each association and whether it is statistically significant.

All 95% confidence intervals depicted within each horizontal line correspond with a given risk factor; the odds ratio point estimates are marked with red dots. The vertical dashed line at 11 is the threshold for no association, meaning that risk factors with confidence intervals cross this line are not statistically significant.

There was a strong and statistically significant association between smoking status and risk for bladder cancer, such that fixed intervals from all specific factors are wholly to the right of that dashed line, indicating an odds ratio greater than 1.0.

Likewise, there is significant associational evidence for occupational carcinogen exposure and past infection history; their relative interval does not cross the criterion threshold. By contrast, BMI and menopausal status have confidence intervals spanning the vertical line, implying weak evidence that they have any other role in the risk of developing bladder cancer. Aside from these findings, no further evidence speaks in favor of independent predictors of bladder cancer within this population.

## Discussion

This study looks at some of the factors that increase the risk of bladder cancer in women, including smoking, occupational exposure, recurrent urinary tract infections, body mass index, menopausal status, and family history of cancer. Based on a large multi-center cohort and modern statistical methods, this study advances ideas about women’s unique risk profiles for bladder cancer. The results corroborate the previously established risk factors and suggest the potential interactions of hormonal status with other risk factors that may also contribute to the development of bladder cancer.

### Smoking and occupational exposure

Our results indicate smoking as one of the significant risk factors for bladder cancer among women, indicative of an odds ratio of 2.15 and confirming past studies of a strong association between smoking and bladder cancer risk (1, 2). The role of smoking in carcinogenesis is well documented, and some of its carcinogens are excreted through urine, affecting the urothelial lining of the bladder and promoting its cancer development. Our study presents additional data that post-menopausal women appear to have a relatively enhanced risk from smoking, which may be stamped by hormonal changes associated with estrogen deficiency. Hence, this adds credence to earlier studies indicating estrogen’s protective role concerning the bladder’s urothelial integrity (3–5).

Occupational exposure to carcinogens such as aromatic amines and polycyclic aromatic hydrocarbons was found to be another significant risk factor, with women exposed to industrial chemicals being nearly twice as likely to develop bladder cancer (OR = 1.89). This is associated with other studies highlighting prolonged exposure to chemicals in dye production and rubber manufacturing as core risk factors for bladder cancer (6). The study shows the need to recognize women’s occupational hazards in industrial environments. It demonstrates that women in these settings may have similar exposures to carcinogens as their male counterparts. Given these findings, there is a pressing demand for revised workplace safety regulations that address the unique risks associated with women laboring in such industries (7, 8).

### Recurrent UTIs and bladder cancer risk

There is increasing evidence linking recurrent UTIs to risk for the development of bladder cancer, with an odds ratio of 1.72. Chronic inflammation is known to lead to tumorigenesis by mechanisms such as DNA methylation, histone modification, and altered gene expression (9, 13, 14). Women are especially vulnerable to recurrent UTIs because of anatomical predisposing factors, which may, in turn, confer an increased risk of bladder cancer. Our findings indicate that recurrent UTIs may qualify as one of the most important yet least cautioned modifiable risk factors for bladder cancer in women.

### Menopausal status

Interestingly, this study did not find menopausal status to be an independent predictor for bladder cancer. However, modification of the effects of smoking and recurrent UTIs was profound. The subgroup analysis indicated that post-menopausal women who were exposed to either smoking or recurrent UTIs were at greater risk of developing bladder cancer than pre-menopausal women. This suggests that hormonal changes, particularly the decline in estrogen, could further enhance the carcinogenic effects of smoking and chronic inflammation. Estrogens are thought to protect the urothelium, and their decline after menopause could make the bladder more vulnerable to the damaging effects of carcinogens or chronic inflammation (12, 15). Evidence for estrogen as protective is met with reports to the contrary, exemplifying the gaps that exist in our understanding of how hormonal changes happening during menopause interact with life and environmental factors to influence bladder cancer risk (13–19).

### BMI and family history

Against the background of other studies, the present cohort seems to show no remarkable correlation between BMI and bladder cancer risk. In this regard, obesity maintains some association with different cancers due to certain underlying factors like chronic inflammation and insulin resistance. Our findings suggest that BMI may have little impact on bladder cancer risk among women (18, 20). This is in line with other studies, which have also found nonconclusive data between obesity and bladder cancer risk.

Likewise, a family history of cancer was not revealed to be a statistically significant predictor of Bladder cancer in our cohort. Given that family history is a well-established risk factor for many cancers, the fact that it was unassociated with bladder cancer in this study suggests that environmental and lifestyle influences rather than genetic predisposition exert a more significant impact on bladder cancer (21, 22). These findings highlight the significant role of lifestyle factors that can be modified, such as smoking and recurrent UTIs, in the prevention of Bladder cancer.

### Genetic and epigenetic factors

Recent studies have highlighted the role of epigenetic changes, such as DNA methylation and histone modifications, in developing bladder cancers. The multifactorial nature of bladder cancer encompasses various interplaying genetic and environmental components, coupled with the involvement of epigenetic changes in the advancement of the disease (23–25). This study suggests that non-genetic risk factors, such as smoking and recurrent UTIs, may incite epigenetic changes that predispose to the development of bladder cancer. This opens up a larger avenue for research on both genetic and epigenetic causatives of bladder cancer to understand its development further and possibly discover several therapeutic targets.

### Microbiota and cancer risk

Emerging evidence has suggested that the gut and bladder microbiota are relevant to the risk of such carcinomas, including that of the bladder, for some time. The dysbiosis of the bladder microbiota has been linked to the development of cancer itself, as it can affect the immune response or promote carcinogenesis. Another line of recent studies presents microbiota-based interventions and microbiota-directed therapies in targeted approaches to reduce bladder cancer incidence (24, 26–32). This is an exciting area of research that would present preventive strategies and treatment approaches toward bladder carcinoma, especially for women at greater risk owing to lifestyle and hormonal factors (33).

### Study limitations

The current study provides relevant information regarding the risk factors related to bladder cancer among women and has several limitations. First, it was a retrospective study susceptible to recall and selection biases. The cohort barriers came from one region, with certain variables such as ethnically diverse distribution not fully covered, limiting the generalization of how far these findings can be extrapolated. In addition, though we used propensity score matching, thus balancing many baseline covariates to reduce the effects of any baseline imbalance, other unmeasured confounders may also have impacted our results (34). Future studies might better rule out these issues by using more diverse populations with longitudinal data to validate these observations and better understand the complicated interplays of genetic, epigenetic, and environmental factors.

### Future directions

This study has certainly added to the body of knowledge surrounding bladder cancer risk factors in women, but several important areas should be followed up on. Future studies must assess the role of epigenetic changes in DNA methylation and histone modification that may influence bladder cancer development and risk (35). More long-term studies are needed to handle better the temporal relationships between lifestyle factors, hormonal changes, and cancer risk.

There is also a need for deeper exploration of the roles of microbiota in bladder cancer. Looking at the bladder and gut microbiomes may offer some novel avenues for prevention, particularly in the direction of microbiota-based interventions.

Finally, for the generalizability of bladder cancer risk models derived from findings in this study, the extension of the survey to include various ethnic communities and geographical regions in the future is posited to validate these findings (36).

## Conclusion

In this study, smoking, occupational exposure, and recurrent UTIs were identified as the principal causes of bladder cancer in women. Menopausal status was discovered to moderate the influence of smoking and UTIs. Although BMI and family history of cancer were not significant predictors in this cohort, the observations give prominence to gender-specific prevention and screening. Future studies should elucidate biological mechanisms underlying the association; significantly, the interaction of hormonal status and traditional risk factors will benefit the prevention and treatment of women.

## Data Availability

The original contributions presented in the study are included in the article/supplementary material. Further inquiries can be directed to the corresponding author.
